# Adding Modified Buckwheat Sprouts to an Atherogenic Diet — the Effect on Selected Nutritional Parameters in Rats

**DOI:** 10.1007/s11130-023-01047-9

**Published:** 2023-02-06

**Authors:** Marta Molska, Julita Reguła, Michał Świeca

**Affiliations:** 1 Department of Human Nutrition and Dietetics, Faculty of Food Sciences and Nutrition, Poznan University of Life Sciences, 28 Wojska Polskiego Street, 60-624 Poznan, Poland; 2 Department of Food Chemistry and Biochemistry, University of Life Sciences in Lublin, Skromna Str. 8, 20-704 Lublin, Poland

**Keywords:** Apparent digestibility, Morphological analysis, *Fagopyrum esculentum* Moench, *Saccharomyces cerevisiae* var. *boulardii*, Pseudocereals

## Abstract

The germinated seeds of many plants are a natural source of substances that can be used to supplement food and increase its functionality. The seeds’ metabolism may be modified during germination to produce specific health-promoting compounds. *Fagopyrum esculentum* Moench is a rich source of nutrients. Buckwheat seeds modified during germination may be helpful as an additive to new functional food products with anti-atherogenic properties. However, their effect and safety should be assessed in *in vivo* studies. The aim of the study was to evaluate the effect that adding modified buckwheat sprouts (*Fagopyrum esculentum* Moench) to an atherogenic (high-fat) diet has on the morphology and digestibility parameters of rats. Buckwheat seeds were modified by adding the probiotic strain of the yeast *Saccharomyces cerevisiae* var. *boulardii*. The study was carried out on 32 Wistar rats, and digestibility and blood counts were assessed during the experiment. There was no evidence of an adverse effect on the animals' weight gain and nutritional efficiency. However, the influence of diets with freeze-dried buckwheat sprouts on digestibility and morphological parameters was noticed. Fat digestibility registered a statistically significant decrease in the groups fed a high-fat diet with the addition of sprouts. The study shows a new direction in the use of buckwheat sprouts.

**Supplementary Information:**

The online version contains supplementary material available at 10.1007/s11130-023-01047-9.

## Introduction

Buckwheat is a crucial pseudocereal produced in many countries, such as Japan, China, Russia, and European countries. *Fagopyrum esculentum* Moench, or common buckwheat, is a good source of pro-health ingredients, especially protein, bioactive compounds such as flavonoids, flavones, phenolic acids, tannins, phytosterols and phagopyrins, which can be found in the grain and husk of buckwheat [1]. It is usually used in grains and ground form, which is processed into other food products, such as pasta and bread. Furthermore, sprouting is an integral part of the cultivation process, resulting in sprouts with a nutritional value different from the seeds mentioned above [1, 2].

Sprouting is a method of improving a product's nutritional value and functionality. During this process, several changes occur in the raw material. For example, Yiming et al. [1] showed that the content of total flavonoids increased during the sprouting process. However, other studies have shown that fats, proteins, and starch are broken down into compounds that are a source of energy and substrates from which newly synthesized compounds are formed [1, 3–5].

The search for new functional products sets the trend for the industry and creates the need to check and confirm their pro-health effects. One of the products that can be used as an element of functional food are pseudocereals. Due to their excellent nutritional value, they have been referred to as "grains of the twenty-first century". They are rich in protein with a high biological value, starch, trace elements or a group of bioactive ingredients (saponins, phenolic compounds, phytosterols, phytoecdysteroids, polysaccharides, betalains and bioactive proteins and peptides). They also have a well-balanced amino acid composition [6–8].

Buckwheat protein was found to be poorly digestible, despite its high biological value. The poor digestibility and thus availability of buckwheat proteins is mainly caused by the high level of protease inhibitors (substances that inhibit the activity of proteolytic enzymes) and tannins (vegetable tannins, organic chemical compounds). A special feature of buckwheat kernels is the specific combination of phenolic compounds with proteins, which may reduce their enzymatic availability. One of the methods of improving digestibility may be the partial reduction of tannins, flavonoids or phenolic acids due to roasting and thermal deactivation of trypsin inhibitors [8–13].

Digestibility is essential in interpreting the quality of the nutrients the body digests. In practice, this is the difference between the amount of nutrient ingested and the amount produced in the stools. Food processing alters molecular, supramolecular structures, allowing digestive enzymes to access nutrients more easily. Consequently, it should improve the digestibility of the food. Germination or technological changes of grains create opportunities to obtain new sources of functional food rich in health-promoting substances. The nutritional composition of a sprout depends on factors such as the type of sprout and the germination conditions. Many biochemical changes occur in the germination process, thus affecting the nutritional value of the raw material. During this process, a large number of hydrolytic enzymes are synthesized or released, which degrade anti-nutritional factors or hydrolyze what might be termed biopolymers, *e.g*., proteins [5, 10, 14, 15].

On the other hand, adding sprouts to a product, for example, may change its nutritional value. Alvarez-Jubete et al. [16] observed that the baking process had a negative effect on the antioxidant properties of bread. Bread with the addition of buckwheat sprouts had a much higher total phenol content as well as a higher antioxidant capacity than the wheat bread used as a control [16]. In another study, adzuki bean sprouts and lentils were enriched and used as carriers for *Saccharomyces cerevisiae* var. *boulardii*. The authors found that the resulting sprouts enriched with *S. cerevisiae* var. *boulardii* were a new functional food product [17].

The yeast *Saccharomyces cerevisiae* is a very good model system for studying the function-gene relationship,* e.g*., in fatty acid metabolism, as Trotter [18] reports. Fatty acids are broken down by yeast only in peroxisomes. In contrast, genes encoding core as well as helper peroxisomal *β*-oxidation enzymes have recently been identified. The *de novo* fatty acid profile of yeast is characterized by a majority of saturated and monoenoic acids (containing 16 and 18 carbon atoms). However, approximately 1-2% of total fatty acids synthesized are fatty acids containing 20–30 carbon atoms [18, 19]. Modifying seeds by adding a probiotic yeast strain had a beneficial effect on the change in fatty acid composition (reduction in saturated fatty acids in favor of polyenic fatty acids) [20, 21]. It can also change the bioavailability of ingredients, as well as their quantity, *e.g*., protein [22].

Taking into account the above information, the following hypotheses were put forward: 1/ the modification of sprouts affects the digestibility of selected macronutrients and dry matter; 2/ a diet with probiotic-rich sprouts affects the nutritional parameters of the rats fed with it.

The aim of the study was to evaluate the effect of adding modified buckwheat sprouts (*Fagopyrum esculentum* Moench) to an atherogenic (high-fat) diet on the morphology and digestibility parameters of rats. Buckwheat seeds have been modified by adding the probiotic strain of the yeast *Saccharomyces cerevisiae* var. *boulardii*.

## Materials and Methods

The material and methods section is presented as supplementary material 1.

## Results and Discussion

Table 1S (in Supplementary Material 1) shows the energy and nutritional value of the dry matter in the experimental diets. The AIN-93M diet had the highest energy value (2.28 MJ/100 g), and the high-fat diet (1.85 MJ/100 g) had the lowest. Protein content was highest in the AIN-93M group, followed by HFDPRS > HFD > HFDCS. However, the AIN-93M diet was characterized by 70.1% lower fat content compared to the HFD diet.

The HFD, or a high-fat (atherogenic) diet, is a modified AIN-93M diet containing the addition of lard. Lard was added to the diet to induce inflammation in the rat's body. The addition of lard to an atherogenic diet may cause inflammation in rats, as confirmed by Molska et al. [21]. The CRP index in the group with the addition of lard was higher than in the AIN control group [21]. Overall, a high-fat diet triggered the development of metabolic syndrome, which includes oxidative stress, the onset of atherogenic dyslipidemia, pro-inflammatory and pro-thrombotic states, high blood pressure, central obesity, and cardiovascular disease [23].

Energy value, fat, protein, and carbohydrate content were statistically significant different between all diets. The addition of lard increased the amount of fat in the high-fat diets compared to the AIN-93M diet. The HFDPRS diet was characterized by a statistically lower content of carbohydrates compared to the HFDCS diet. It was found that the addition of sprouts increased the carbohydrate content in the groups fed with the HFDCS and HFDPRS diets compared to the group fed with the HFD diet. Noticeable differences in the carbohydrate content in the diets tested could result from yeast activity, which can break down starch [24]. In Molska et al. [21], starch content in modified buckwheat sprouts was lower than in the control sprouts. However, what should be emphasized is that the amount of resistant starch was higher [22].

**Fig. 1:**
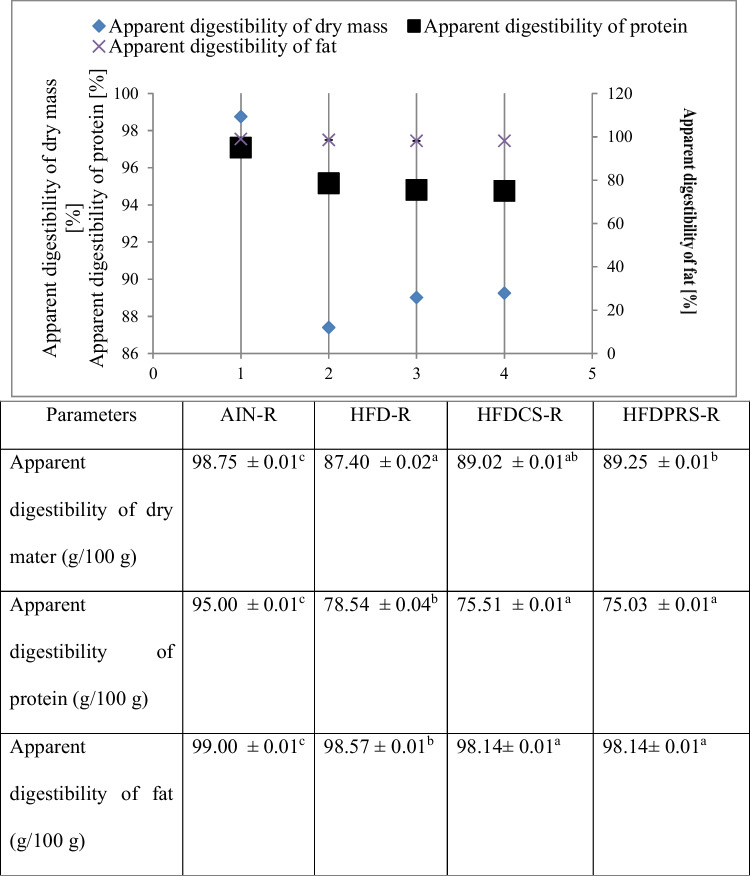
Determination of digestibility indexes. Data are mean ± standard deviation. Values with the same superscript letter in each row are not significantly different (*P* ≤ 0.05). 1: AIN-R; 2: HFD-R; 3: HFDCS-R; 4: HFDPRS-R; AIN-R: a group of rats fed the AIN-93M diet; HFD-R: the group fed the HFD diet; HFDCS-R: a group of rats fed the HFDCS diet; HFDPRS-R: a group of rats fed the HFDPRS diet

The type of nutrition has a significant impact on the nutritional status. In the present study, the digestibility of rats was assessed between 19 and 28 days and the data obtained are presented in Table 1 and Fig. 1.

**Table 1:** Nutritional parameters of animals fed experimental diets

Parameters	Groups of rats			
	AIN-R	HFD-R	HFDCS-R	HFDPRS-R
Initial body weight on the beginning of digestibility (g)	266.13 ± 31.67^b^	255.63 ± 20.84^ab^	289.75 ± 21.51^a^	292.13 ± 16.15^b^
Food intake (g/10 days)	207.08 ± 16.78^b^	170.64 ± 15.00^a^	184.84 ± 8.10^a^	184.83 ± 4.40^a^
Energy intake (MJ/10 days)	3.69 ± 0.29^a^	3.65 ± 0.32^a^	3.76 ± 0.16^a^	3.78 ± 0.09^a^
FER	0.17 ± 0.05^a^	0.17 ± 0.03^a^	0.18 ± 0.03^a^	0.18 ± 0.03^a^
Weight-to-length ratio (g/cm)	12.82 ± 0.81^ab^	12.00 ± 0.87^a^	13.39 ± 0.73^b^	13.46 ± 0.40^b^
Body weight gain (g/10 days)	36.25 ± 13.30^a^	29.63 ± 7.10^a^	32.50 ± 6.00^a^	33.63 ± 5.83^a^
Fecal excretion (g/10 days)	8.81 ± 0.57^a^	24.40 ± 4.15^b^	26.08 ± 3.20^b^	23.65 ± 1.03^b^
Fecal excretion (g dry matter/10 days)	6.15 ± 0.44^a^	20.20 ± 3.12^b^	20.23 ± 2.65^b^	19.75 ± 0.76^b^
Water excretion in feces (g/10 days)	30.00 ± 0.01^c^	16.66 ± 0.01^a^	21.99 ± 0.04^b^	16.38 ± 0.02^a^
Passage time (min)	791.50 ± 111.00^a^	687.50 ± 28.00^a^	774.00 ± 125.00^a^	752.50 ± 111.00^a^

Data are mean ± standard deviation. Values with the same superscript letter in each row are not significantly different (*P* ≤ 0.05). *AIN-R* A group of rats fed the AIN-93M diet; *FER* Food efficiency ratio; *HFD-R* The group fed the HFD diet; *HFDCS-R* A group of rats fed the HFDCS diet; *HFDPRS-R* A group of rats fed the HFDPRS diet

All the rats were weighed at the beginning of the 10-day digestibility study period. The rats that consumed the HFD diet had the lowest body weight, followed by those in the AIN-93M-R, HFDCS-R, and HFDPRS-R groups, respectively. The greatest weight gain was observed in the AIN-93M-R control group, followed by the HFDPRS-R, HFDCS-R and HFD-R groups. During the experiment, rat feces were collected daily, and at the end of the experiment, it was weighed and dried. It was found that the smallest amount of fecal dry matter was excreted by rats consuming the AIN-93M diet. This value was 68.86% lower than the rats consuming the HFDPRS diet. In the case of fecal water content, the highest water content was found in the faeces of rats consuming the diet without any additives, and the lowest in rats consuming the HFDPRS diet (45.40% lower value).

A statistically significant difference in dietary consumption was observed between the AIN-93M-R group and the other groups. However, there were no statistically significant differences between the consumption of individual high-fat diets (HFD, HFDCS, HFDPRS).

The energy from the diet consumed was comparable between the study groups and ranged from 3.65–3.78 MJ/10 days of consumption. An intestinal transit study was carried out during the experiment. The times for the individual groups AIN-93M-R, HFD-R, HFDCS-R and HFDPRS-R are presented in Table 1. Feeding the rats a diet with the addition of lyophilisate control sprouts or probiotic-rich sprouts had no adverse effect on body weight gain or food efficiency. On the other hand, a higher ratio of body weight to length was noted in the rats in the groups supplemented with sprouts (Table 1).

Clinical trials and mechanistic studies on isolated as well as extracted fibers showed a promising regulatory effect on the gut (*e.g*., digestion and absorption, transit time, stool formation) [25]. The publications contain information showing the increase in stool weight significantly reduces its passage time [26]. This may be due to the presence of dietary fiber in the raw material. In this study, such a situation was noted. Compared to the AIN-93M-R group, where the total amount of feces was 8.81 g/10 days, the passage in the HFD-R group was significantly shorter and the total amount of feces was 24.4 g/10 days. In diets with lyophilisate of sprouts, the passage time was shorter than in the AIN-R group. The amount of feces in these diets was 26.08 g/10 days in the HFDCS-R group and 23.65 g/10 days in the HFDPRS-R group. The faster movement of undigested food through the gastrointestinal tract reduces the contact with the intestinal walls, reduces the time of action of digestive enzymes, and may also reduce the risk of colorectal cancer, for example. Such a protective effect is demonstrated by the soluble fraction of dietary fiber, a component found in buckwheat. The dietary fiber content is significantly higher in buckwheat seeds compared to amaranth and quinoa, which have fiber levels comparable to those in regular cereals. According to the literature data on Tartary buckwheat, the total content of dietary fiber (TDF) in seeds is 26%, and soluble (SDF) and insoluble (IDF) 0.54% and 24%, respectively [27–29].

In order to characterize the biological properties of probiotic-rich sprouts, their effect on laboratory animals was analyzed based on selected digestibility indexes. Protein digestibility was 95.00 ± 0.01 (%) in the group fed with the AIN-93M diet. In subsequent groups, the values were, respectively: 78.54 ± 0.04% (HFD-R), 75.51 ± 0.01% (HFDCS-R), 75.03 ± 0.01% (HFDPRS-R). There are statistically significant changes between the food groups of diets with added sprouts and HFD and AIN-93M. Protein digestibility in the diets supplemented with sprouts decreased by 19.49 g/100 g (HFDCS) and 19.97 g/100 g (HFDPRS) compared to the AIN-93M diet.

Fat digestibility is lower in the groups with the addition of sprouts compared to the AIN-93M and HFD diets (statistically significant difference).

Table 2 shows the effect of feeding rats with a diet with the addition of lyophilisates of sprouts on blood morphological parameters. There were slight changes in the levels of these parameters.

**Table 2:** Influence of the diets with or without probiotic-rich sprouts on morphological parameters in rats

Parameters	Groups of rats			
	AIN-R	HFD-R	HFDCS-R	HFDPRS-R
WBC G/l	5.70 ± 1.60^b^	4.18 ± 1.00^ab^	3.91 ± 0.94^a^	4.10 ± 1.30^ab^
NEU G/l	0.44 ± 0.08^a^	0.34 ± 0.10^a^	0.33 ± 0.07^a^	0.32 ± 0.12^a^
LYM G/l	5.12 ± 1.67^b^	3.45 ± 0.70^ab^	3.37 ± 0.87^a^	3.63 ± 1.19^ab^
MONO G/l	0.21 ± 0.07^b^	0.19 ± 0.07^ab^	0.17 ± 0.07^ab^	0.11 ± 0.04^a^
EOS G/l	0.01 ± 0.02^a^	0.01 ± 0.01^a^	0.01 ± 0.01^a^	0.02 ± 0.01^a^
BASO G/l	0.06 ± 0.02^c^	0.02 ± 0.02^a^	0.03 ± 0.03^ab^	0.05 ± 0.02^bc^
RBC T/l	7.89 ± 0.14^b^	7.47 ± 0.25^a^	7.50 ± 0.33^a^	7.53 ± 0.30^a^
HGB g/l	146.00 ± 4.41^b^	139.88 ± 4.22^ab^	137.50 ± 5.01^a^	139.25 ± 4.83^a^
HCT l/l	0.47 ± 0.02^b^	0.45 ± 0.01^ab^	0.44 ± 0.02^a^	0.45 ± 0.02^ab^
MCV fl	59.28 ± 1.69^a^	60.76 ± 1.44^a^	59.23 ± 1.53^a^	59.6 ± 1.14^a^
MCH pg	18.51 ± 0.40^a^	18.74 ± 0.55^a^	18.35 ± 0.49^a^	18.51 ± 0.53^a^
MCHC g/l	312.25 ± 3.54^a^	308.13 ± 6.24^a^	309.75 ± 3.54^a^	310.75 ± 5.90^a^
PLT G/l	862.38 ± 174.61^a^	835.17 ± 95.09^a^	891.00 ± 143.59^a^	926.86 ± 70.80^a^

Data are mean ± standard deviation. Values with the same superscript letter in each row are not significantly different (*P* ≤ 0.05). *AIN-R* A group of rats fed the AIN-93M diet; *BASO* Basophils; *EOS* Eosinophils; *HCT* Hematocrit; *HFD-R* The group fed the HFD diet; *HFDCS-R* A group of rats fed the HFDCS diet; *HFDPRS-R* A group of rats fed the HFDPRS diet; *HGB* Hemoglobin; *LYM* Lymphocytes; *MCH* Mean blood hemoglobin concentration; *MCHC* Mean blood hemoglobin; *MCV* Mean red blood cell volume; *MONO* Monocytes *NEU* Neutrophils; *PLT* Thrombocytes; *RBC* Red blood cells; *WBC* White blood cells

In the case of rats in the AIN-R group, most morphological parameters were lower in the HFD-R, HFDCS-R, and HFDPRS-R groups. Morphology allows the health status of rats to be assessed. It focuses primarily on the system of red, white blood cells, platelets and the so-called erythrocyte indices. In the HFDPRS-R and HFDCS-R groups, compared to the HFD-R and AIN-93M groups, a decrease was noted in the WBC index, which indicates the number of leukocytes. This may suggest, inter alia, stress and malnutrition.

Changes in the number of red blood cells and hemoglobin in the groups fed the atherogenic diet with the AIN-93M diet may indicate possible anemia in the rats. Taking into account the information presented above, it should be noted that the addition of the lyophilisate of probiotic sprouts could be a factor that improved the values of morphological parameters in the HFDPRS-R group, compared to the rats fed a high-fat diet (HFD-R).

## Conclusion

These results show that feeding the rats an atherogenic diet with the addition of probiotic-rich sprouts did not affect weight gain or nutritional efficiency. In the parameters of fat digestibility, there are statistically significant differences between rats fed with the addition of sprouts and those fed with AIN-93M and HFD diets. In addition, diet with lyophilized probiotic-rich sprouts influenced the values of morphological parameters.

Therefore, research should be extended to confirm the impact of modified buckwheat sprouts on digestibility, *e.g*., in humans. The current trend of searching for new functional raw materials offers many opportunities for both technologists and dieticians. This research gives a new direction for the use of buckwheat sprouts as the raw material.

## Supplementary Information

Below is the link to the electronic supplementary material.

Supplementary file1 (DOCX 34.1 KB)

## Data Availability

Not applicable.

## Abbreviations

AIN-93M: Standard diet
AIN-R: A group of rats fed the AIN-93M diet
BASO: Basophils
EOS: Eosinophils
HCT: Hematocrit
HFD: High-fat diet
HFD-R: The group fed the HFD diet
HFDCS: Modified AIN-93M diet by adding lard and buckwheat sprouts lyophilisate
HFDCS-R: A group of rats fed the HFDCS diet
HFDPRS: Modified AIN-93M diet by adding lard and modified buckwheat sprouts lyophilisate
HFDPRS-R: A group of rats fed the HFDPRS diet
HGB: Hemoglobin
LYM: Lymphocytes
MCH: Mean blood hemoglobin concentration
MCHC: Mean blood hemoglobin
MCV: Mean red blood cell volume
MONO: Monocytes
NEU: Neutrophils
PLT: Thrombocytes
RBC: Red blood cells
WBC: White blood cells

## References

[1] Yiming Z, Hong W, Linlin C (2015). Evolution of nutrient ingredients in tartary buckwheat seeds during germination. Food Chem.

[2] Suzuki T, Hara T, Katsu K (2021). Breeding of buckwheat for usage of sprout and pre-harvest sprouting resistance. Plants.

[3] Benincasa P, Falcinelli B, Lutts S (2019). Sprouted grains: a comprehensive review. Nutrients.

[4] Krumina-Zemture G, Beitane I (2017) Fatty acid composition in buckwheat (*Fagopyrum esculentum* M.) flours and their extruded products. Proceedings of the 8th International Scientific Conference Rural Development, Kaunas, Lithuania, pp 66–69. 10.15544/RD.2017.017

[5] Zhang G, Xu Z, Gao Y (2015). Effects of germination on the nutritional properties, phenolic profiles, and antioxidant activities of buckwheat. J Food Sci.

[6] Martínez-Villaluenga C, Peñas E, Hernández-Ledesma B (2020). Pseudocereal grains: nutritional value, health benefits and current applications for the development of gluten-free foods. Food Chem Toxicol.

[7] Zawadzka A, Kobus-Cisowska J, Stachowiak B (2021). Bioactive metabolites of buckwheat (*Fagopyrum* Mill.). Zag Doradz Rol.

[8] Zhou M-L, Wieslander G, Tang Y et al (2016) Chapter eleven - Bioactive compounds in buckwheat sprouts. In: Zhou M, Kreft I, Woo S-H, et al (eds) Molecular breeding and nutritional aspects of buckwheat. Academic Press, pp 151–159. 10.1016/B978-0-12-803692-1.00011-0

[9] Christa K, Soral-Śmietana M (2008) Effect of roasting process on the enzymatic digestibility of proteins in grains of buckwheat (*Fagopyrum esculentum * Moench). Żywn Nauka Technol Jakość 5:52–62 [Polish]

[10] Joye I (2019). Protein digestibility of cereal products. Foods.

[11] Tomotake H, Kayashita J, Kato N (2015). Hypolipidemic activity of common (*Fagopyrum esculentum* Moench) and tartary (*Fagopyrum tataricum* Gaertn.) buckwheat: hypolipidemic activity of buckwheat. J Sci Food Agric.

[12] Zarzecka K, Gugała M, Mystkowska I (2014). Nutritional value and opportunities of using buckwheat. Post Fitoter.

[13] Zhang Z-L, Zhou M-L, Tang Y (2012). Bioactive compounds in functional buckwheat food. Food Res Int.

[14] Lawrence JM, Lawrence AL, Watts SA (2020) Chapter 9 - Ingestion, digestion, and digestibility of regular sea urchins. In: Lawrence JM (ed) Developments in aquaculture and fisheries science. Elsevier, pp 165–190. 10.1016/B978-0-12-819570-3.00009-3

[15] Miyahira RF, de Oliveira Lopes J, Antunes AEC (2021). The use of sprouts to improve the nutritional value of food products: a brief review. Plant Foods Hum Nutr.

[16] Alvarez-Jubete L, Arendt EK, Gallagher E (2009). Nutritive value and chemical composition of pseudocereals as gluten-free ingredients. Int J Food Sci Nutr.

[17] Swieca M, Kordowska-Wiater M, Pytka M et al (2019) Nutritional and pro-health quality of lentil and adzuki bean sprouts enriched with probiotic yeast * Saccharomyces cerevisiae * var. * boulardii*. LWT-Food Sci Technol 100:220–226. 10.1016/j.lwt.2018.10.081

[18] Trotter PJ (2001). The genetics of fatty acid metabolism in *Saccharomyces cerevisiae*. Annu Rev Nutr.

[19] Eisenberg T, Büttner S (2014). Lipids and cell death in yeast. FEMS Yeast Res.

[20] Molska M, Reguła J, Rudzińska M, Świeca M (2020). Fatty acids profile, atherogenic and thrombogenic health lipid indices of lyophilized buckwheat sprouts modified with the addition of *Saccharomyces cerevisiae* var. *boulardii*. Acta Sci Pol Technol Aliment.

[21] Molska M, Reguła J, Grygier A (2022). Effect of the addition of buckwheat sprouts modified with the addition of *Saccharomyces*
*cerevisiae* var. *boulardii* to an atherogenic diet on the metabolism of sterols, stanols and fatty acids in rats. Molecules.

[22] Molska M, Reguła J, Zielińska-Dawidziak M (2022). Starch and protein analysis in buckwheat (Fagopyrum esculentum Moench) sprouts enriched with probiotic yeast. LWT-Food Sci Technol.

[23] Lasker S, Rahman MM, Parvez F (2019). High-fat diet-induced metabolic syndrome and oxidative stress in obese rats are ameliorated by yogurt supplementation. Sci Rep.

[24] Mousa A, Bakry A, Wang G, Zhang H (2019). Efficacy of *Saccharomyces boulardii* metabolism during fermentation of milk fortified with wheat grain juice. Food Sci Technol Res.

[25] Gill SK, Rossi M, Bajka B, Whelan K (2021). Dietary fibre in gastrointestinal health and disease. Nat Rev Gastroenterol Hepatol.

[26] Sarriá B, Martínez-López S, Fernández-Espinosa A (2012). Effects of regularly consuming dietary fibre rich soluble cocoa products on bowel habits in healthy subjects: a free-living, two-stage, randomized, crossover, single-blind intervention. Nutr Metab.

[27] Kumar V, Sinha AK, Makkar HPS (2012). Dietary roles of non-starch polysaccharides in human nutrition: a review. Crit Rev Food Sci Nutr.

[28] Sytar O, Biel W, Smetanska I, Brestic M (2018) Bioactive compounds and their biofunctional properties of different buckwheat germplasms for food processing. In: Buckwheat germplasm in the world, 1st edn. Springer, pp 191–204. 10.1016/B978-0-12-811006-5.00019-7

[29] Ocvirk S, Wilson AS, Appolonia CN (2019). Fiber, fat, and colorectal cancer: new insight into modifiable dietary risk factors. Curr Gastroenterol Rep.

