# Supplementary material for: Adding Modified Buckwheat Sprouts to an Atherogenic Diet — the Effect on Selected Nutritional Parameters in Rats
Source: Plant Foods Hum Nutr. 2023 Feb 6;78(2):279–85. doi: 10.1007/s11130-023-01047-9 (PMC10363063; doi:10.1007/s11130-023-01047-9)
Supplement: Supplementary file 1 — Supplementary file1 (DOCX 34.1 KB) [file 11130_2023_1047_MOESM1_ESM.docx]

**SUPPLEMENTARY MATERIAL**

**Title:** Adding modified buckwheat sprouts to an atherogenic diet - the effect on selected nutritional parameters in rats

**Journal name**: Plant Foods for Human Nutrition

**Author names:** Marta Molska^1*^, Julita Reguła^1^, Michał Świeca^2,^

^1^Department of Human Nutrition and Dietetics, Faculty of Food Sciences and Nutrition, Poznan University of Life Sciences, 28 Wojska Polskiego Street, 60-637 Poznan, Poland; <https://orcid.org/0000-0002-1514-2103> (M.M.); <https://orcid.org/0000-0002-3265-547X> (J.R.)

^2^ Department of Food Chemistry and Biochemistry, University of Life Sciences in Lublin, Skromna Str. 8, 20-704 Lublin, Poland; <https://orcid.org/0000-0002-6513-8399> (M.Ś.)

* To whom correspondence should be addressed

Mailing address: Department of Human Nutrition and Dietetics, Faculty of Food Sciences and Nutrition, Poznan University of Life Sciences, 28 Wojska Polskiego Street, 60-637 Poznan, PolandE-mail: [marta.molska@up.poznan.pl](mailto:marta.molska@up.poznan.pl). Phone number:+48618487594 (MM).

**Material and methods:**

**Buckwheat grains and preparation of control and probiotic-rich sprouts**

*Fagopyrum esculentum* Moench seeds were obtained from PNOS SA in Ożarów Mazowiecki, Poland. The *Saccharomyces cerevisiae* var *boulardii* strain was grown on malt agar (at 30 °C for 48 hours). In the next step, the colonies were sterile-picked and then suspended in water. Part of the buckwheat seeds was placed in the inoculum prepared in this way. The other part of the buckwheat seeds was placed in distilled water for 4 hours (control). After disinfecting the seeds with 1% sodium hypochlorite, they were drained and washed with distilled water. In the next step, the seeds germinated for three days in the dark, having been placed in a growth chamber. Then, they were harvested and sprayed with Milli-Q water. The seeds and sprouts were lyophilized and frozen for analysis. The material was prepared according to the method described in Molska et al. (2020) [1].

**Animals**

Animal procedures were approved by the local bioethics committee (approval number 28/2017). Thirty-two 8-week-old male Wistar albino rats were used in this study and the mean weight of the animals was 188.7 ± 14.1g The rats were housed on a 12-hour light/dark cycle with a thermostatic control (20°C±2.0) and 55-65% humidity for both the adaptation and experimental periods [2].

**Experimental Design**

Before starting the experiment, the animals underwent a period of adaptation to laboratory conditions (three days). The rats had free access to water and a basic diet of AIN-93M during this period. In addition, animals were housed in metal-free enamel-coated stainless steel cages during the adaptation and experiment periods [2].

After this adaptation period, the rats were randomized into four groups of eight individuals each. There were two rats in each cage. One of the groups was fed with the AIN-93M diet; the next three groups were fed with the modified AIN-93M diet with the addition of lard in the amount of 200 g/kg of the diet (HFD, HFDCS, HFDPRS) [3]. The HFDCS and HFDPRS diets included the addition to the high-fat diet (1 kg) of 300 grams of control and modified sprout lyophilisate, respectively. Semisynthetic diets comprised casein (from Murowana Goślina, Poland), soybean oil (ZPT Warszawa, Poland), sucrose (Diamant, Pfeifer & Langen Polska S.A., Poznań, Poland), wheat starch (Celiko, Poznań, Poland), potato starch (potatoes from Iława, Poland), a mineral mix (AIN-93M-MX) [3], a vitamin mix (AIN-93-VX) [3] and choline (Sigma-Aldrich). In addition, sprouts in the form of a lyophilisate were added to the HFDCS and HFDPRS diets. The diets were prepared by mixing all the ingredients [2]. The energy and nutritional value of the dry matter in the experimental diets is presented in Table 1S.

**Table 1S** Energy and nutritional value of the dry matter in experimental diets

| Component | Experimental Diets | | | |
| --- | --- | --- | --- | --- |
|  | AIN-93M | HFD | HFDCS | HFDPRS |
| Energy (MJ/100g) | 1.85 ± 0.20^a^ | 2.28 ± 0.39^d^ | 2.15 ± 0.33^b^ | 2.16 ± 0.33^c^ |
| Protein (%) | 18.29 ± 0.10^d^ | 12.38 ± 0.30^b^ | 12.31 ± 0.90^a^ | 12.78 ± 0.30^c^ |
| Fat (%) | 7.15 ± 0.03^a^ | 23.91 ± 0.70^d^ | 19.07 ± 0.40^b^ | 19.34 ± 0.04^c^ |
| Carbohydrates (%) | 71.97 ± 0.20^d^ | 61.58 ± 0.20^a^ | 66.33 ± 0.10^c^ | 65.59 ± 0.05^b^ |
| Crude ash (%) | 2.48 ± 0.20^b^ | 2.05 ± 0.20^a^ | 2.22 ± 0.10^a^ | 2.22 ± 0.05^a^ |

Data are mean ± standard deviation. Values with the same superscript letter in each row are not significantly different (P≤ 0.05).

Each day, the rats were given a fresh ration of food and water. All residues (food and water) were removed the following day. Diet and water consumption were monitored daily and the animals’ body weights were monitored weekly. During the course of the experiment, the digestibility of the animals was determined [2].

**Apparent digestibility**

Apparent digestibility rates were determined within ten days for all animals using the conventional balancing method. All feces were collected for ten days; the faeces were dried immediately after collection at 60°C for 24 hours.

Dry matter, crude fat and protein were determined in the diets as well as in the feces collected, all using standard analytical methods. The dry weight was determined by drying about 1 g of each sample in an oven at 105 °C for 12 hours and weighing. On the other hand, the ash content was determined by burning at 550 °C for the whole day (24 hours). The total fat was determined using the Soxhlet extraction method. Protein content was determined using the Kjeldahl total nitrogen method. Carbohydrates were the difference of 100 minus, the sum of water, protein, fat and ash content [4].

The apparent nutrient digestibility coefficient of the diets was calculated according to the following equation: Apparent digestibility = 100 × (NI − NE)/NI where NI is nutrient intake and NE is nutrient excreted.

On day 11 of the experiment, the time required for chyme to pass through the alimentary tract of the animals was determined using the indicator method, with 1 g chromium(III) oxide/100 g diet within 24-hour periods, according to the method developed by Gobl and Gohl [5].

Using the Atwater equivalents, the energy value of the diet was calculated [6], and the feed efficiency index was calculated on the basis of the cumulative diet consumption and weight gain [7].

**Analysis of Hematological parameters**

Blood was drawn by cardiac puncture. It was collected in sodium heparin tubes (for whole blood for complete blood counts) and in other separate serum tubes (for biochemical parameters). The coagulated blood was allowed to clot for 30 minutes at room temperature. It was then centrifuged at 3600 x g for 15 min. The following morphological parameters were determined: white blood cells (WBC), mean red blood cell volume (MCV), mean blood hemoglobin concentration (MCH), mean blood hemoglobin (MCHC), lymphocytes (LYM), neutrophils (NEU), red blood cells (RBC), monocytes (MONO), thrombocytes (PLT), eosinophils (EOS), basophils (BASO), hemoglobin (HGB), hematocrit (HCT). Using a Sysmex K-1000 hematology analyzer (TAO Medical Electronics Co., Kobe, Japan), morphological index values were determined according to standard procedures.

**Statistical Analysis**

Statistical analysis of the data was performed using Statistica 10 (StatSoft, Tulsa, OK, USA). For statistical analysis, one-way analysis of variance and intergroup differences was used by means of Tukey's HSD post-hoc test with a significance level of P≤0.05. Significant differences were denoted with different superscript letters.

**References:**

1. Molska M, Reguła J, Rudzińska M, Świeca M (2020) Fatty acids profile, atherogenic and thrombogenic health lipid indices of lyophilized buckwheat sprouts modified with the addition of *Saccharomyces cerevisiae* var. *boulardii*. Acta Scientiarum Polonorum Technologia Alimentaria 19:. https://doi.org/10.17306/J.AFS.2020.0866

2. Molska M, Reguła J, Grygier A, et al (2022) Effect of the addition of buckwheat sprouts modified with the addition of *Saccharomyces cerevisiae* var. *boulardii* to an atherogenic diet on the metabolism of sterols, stanols and fatty acids in rats. Molecules 27:4394. https://doi.org/10.3390/molecules27144394

3. Reeves PG, Nielsen FH, Fahey GC (1993) AIN-93 purified diets for laboratory rodents: final report of the american institute of nutrition ad hoc writing committee on the reformulation of the AIN-76A Rodent Diet. The Journal of Nutrition 123:1939–1951. https://doi.org/10.1093/jn/123.11.1939

4. Horwitz W, Latimer GW (2005) Official methods of analysis of AOAC International. AOAC International, Gaithersburg, Md.

5. Gohl B, Gohl I (1977) The effect of viscous substances on the transit time of barley digesta in rats. J Sci Food Agric 28:911–915. https://doi.org/10.1002/jsfa.2740281008

6. Sánchez-Peña M, Márquez-Sandoval F, Ramírez Anguiano A, et al (2017) Calculating the metabolizable energy of macronutrients: a critical review of Atwater’s results. Nutrition Reviews 75:37–48. https://doi.org/10.1093/nutrit/nuw044

7. Lee Y, Kang E-Y, Park M-N, et al (2008) Effects of sn-2 palmitic acid-fortified vegetable oil and fructooligosaccharide on calcium metabolism in growing rats fed casein based diet. Nutrition research and practice 2:3–7. https://doi.org/10.4162/nrp.2008.2.1.3
